# Deep Brain Stimulation: Emerging Tools for Simulation, Data Analysis, and Visualization

**DOI:** 10.3389/fnins.2022.834026

**Published:** 2022-04-11

**Authors:** Karin Wårdell, Teresa Nordin, Dorian Vogel, Peter Zsigmond, Carl-Fredrik Westin, Marwan Hariz, Simone Hemm

**Affiliations:** ^1^Neuroengineering Lab, Department of Biomedical Engineering, Linköping University, Linköping, Sweden; ^2^Institute for Medical Engineering and Medical Informatics, School of Life Sciences, University of Applied Sciences and Arts Northwestern Switzerland, Muttenz, Switzerland; ^3^Department of Neurosurgery and Biomedical and Clinical Sciences, Linköping University, Linköping, Sweden; ^4^Department of Radiology, Brigham and Women’s Hospital, Harvard Medical School, Boston, MA, United States; ^5^Unit of Functional Neurosurgery, UCL Queen Square Institute of Neurology, London, United Kingdom; ^6^Department of Clinical Sciences, Neuroscience, Ume University, Umeå, Sweden

**Keywords:** deep brain stimulation (DBS), modeling and simulation, neuroimaging, probabilistic mapping, connectivity, intraoperative measurements, visualization

## Abstract

Deep brain stimulation (DBS) is a well-established neurosurgical procedure for movement disorders that is also being explored for treatment-resistant psychiatric conditions. This review highlights important consideration for DBS simulation and data analysis. The literature on DBS has expanded considerably in recent years, and this article aims to identify important trends in the field. During DBS planning, surgery, and follow up sessions, several large data sets are created for each patient, and it becomes clear that any group analysis of such data is a big data analysis problem and has to be handled with care. The aim of this review is to provide an update and overview from a neuroengineering perspective of the current DBS techniques, technical aids, and emerging tools with the focus on patient-specific electric field (EF) simulations, group analysis, and visualization in the DBS domain. Examples are given from the state-of-the-art literature including our own research. This work reviews different analysis methods for EF simulations, tractography, deep brain anatomical templates, and group analysis. Our analysis highlights that group analysis in DBS is a complex multi-level problem and selected parameters will highly influence the result. DBS analysis can only provide clinically relevant information if the EF simulations, tractography results, and derived brain atlases are based on as much patient-specific data as possible. A trend in DBS research is creation of more advanced and intuitive visualization of the complex analysis results suitable for the clinical environment.

## Introduction

Implantable stimulation devices are important neuroengineering technologies for improving treatment of neurological and psychiatric disorders and symptoms (Thakor, 2009; Johnson et al., 2013; Ereifej et al., 2019). Deep brain stimulation (DBS) (Benabid et al., 2000; Hariz, 2003; Vissani et al., 2020; Krauss et al., 2021) is one of the most used neurostimulation methods that is well established for movement disorders such as Parkinson’s disease (PD), dystonia, and essential tremor (ET) (Wong et al., 2020). DBS is also being explored for treatment-resistant psychiatric disorders such as obsessive compulsive disorders (OCD) and depression (Hariz et al., 2013; Sullivan et al., 2021).

More than 200,000 devices (Vedam-Mai et al., 2021) have been implanted worldwide, and research in DBS is rapidly gaining interest. An indicator of this is shown in how the number of scientific papers has quadrupled during the past decade. Scientific papers on DBS now number above 17,000 (PubMed December 2021). DBS is truly a multidisciplinary research field involving neurosurgeons, neurologists, neurophysiologists, psychiatrists, ethicists, nurses, and neuroengineers. Clinical studies are often carried out in collaboration with industry. Despite the rise in interdisciplinary collaboration, the scientific output is still mostly dominated by studies with their base in clinical science.

Since DBS implantations require precise and safe targeting of a specific brain structure, stereotactic systems that rely on high quality brain imaging and surgical planning systems are required. Intraoperative recording of physiological signals is used as a complement for target verification and compensation of trajectory deviations due to brain shift (Hemm and Wårdell, 2010; van den Munckhof et al., 2021). With novel DBS lead designs, the postoperative programming of stimulation results in many available options which require the need for support systems. Simulations of electric field (EF) and anatomical brain atlases can help linking DBS-data with patients’ clinical scoring information in the postoperative evaluation sessions. During DBS planning, surgery, and follow up sessions, many data sets are created for one patient. Hence, when group analysis is required, the number of data sets quickly becomes a big data analysis problem.

The aim of this review is to provide an update and overview from a neuroengineering perspective of the present DBS techniques, technical aids, and emerging tools with the focus on simulation, data analysis and visualization in the DBS domain. As it is necessary to be aware of clinical needs for the neuroengineers working with development of methods we give an introduction to DBS systems, clinical indications and the DBS surgical procedure. Due to the diversity and complexity in DBS, we use as starting point methods developed by our consortium for patient-specific EF simulation, data analysis and visualization.

## Deep Brain Stimulation Systems, Clinical Indications, and Brain Targets

Electrical brain stimulation in patients with movement disorders was introduced by Natalia Bechtereva in the mid-1970s (Bechtereva et al., 1975; Blomstedt and Hariz, 2010). Following these successful implantations of gold electrodes in the deep brain structures, the technical development of stimulation devices continued (Coffey, 2009). In 1987, the first modern DBS lead was implanted by Alim Louis Benabid in Grenoble (Benabid et al., 1987; Hariz, 2017), which targeted the nucleus ventrointermedius (VIM) of the thalamus for treatment of tremor. The same team also published, in 1993, the first case of unilateral subthalamic nucleus (STN) DBS in a patient with severe Parkinsonism (Pollak et al., 1993). Later that same year, Lars-Erik Augustinsson performed – but never published – the first bilateral STN implantation in Sweden at Sahlgrenska Hospital, Gothenburg (personal communication). Today, the STN is the most frequently used brain target for DBS in PD. During the first 20 years of the modern DBS era, Medtronic (Minneapolis, MN, United States) was the only DBS company on the market. Today, both Boston Scientific and Abbott (former St Jude) also provide DBS systems which are Conformité Européenne (CE) marked and FDA approved. Recently, the direct STIM™ DBS System, marketed by Aleva Neurotherapeutics, received CE approval.

**FIGURE 1:**
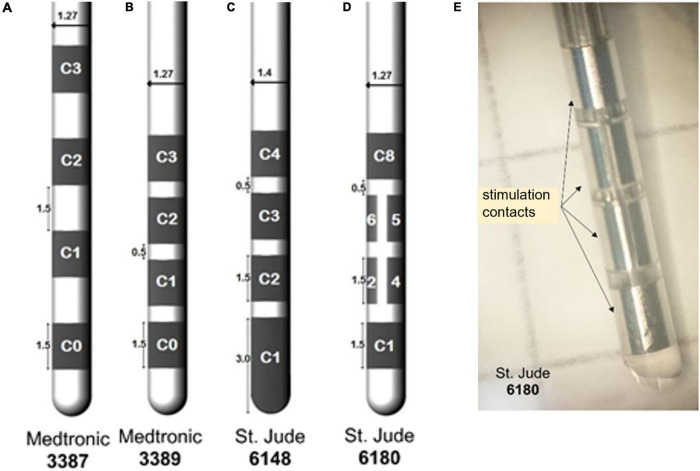
Example of deep brain stimulation (DBS) leads with four contacts and different spacing between the contacts **(A)** 3387, **(B)** 3389, **(C)** tip contact 6148, and **(D)** with segmented ring contact 6180. The numbering of the contacts differs between companies. **(E)** A photo of 6180 with marked stimulation contacts. (Alonso, 2018 with permission).

A variety of DBS lead designs with different contact configurations are available, and examples are shown in Figure 1. Typically, a DBS lead is about 1.3 mm thick and 7.5–10.5 mm long, with four active contacts having a length of 1.5 mm separated by 0.5 or 1.5 mm. There are also configurations with segmented contacts for the steering of the stimulation field in one or more directions. Stimulation is achieved in monopolar or bipolar modes using voltage or current settings. Other modes of stimulation are multiple contact level settings and interleaved, i.e., alternation between contacts and amplitudes. The electric stimulation parameters vary according to the disorders and symptoms to treat. Typical initial settings for PD are 1–4 V in voltage mode and 1–5 mA in current mode at a frequency of 130 Hz and a pulse width of 60 μs (Koeglsperger et al., 2019). However, the frequency and pulse width can be modified during subsequent postoperative clinical evaluations. A recent development by Medtronic is a DBS system designed for combined stimulation and recording of local field potentials (LFP). Using LFP opens for a so-called adaptive stimulation, that is, a closed loop control in the patient’s postoperative management (Thenaisie et al., 2021). The large number of parameter selections can help fine tune the stimulation and thus the clinical outcome, but also makes it more difficult and time-consuming to program a DBS system to optimize the therapy.

**FIGURE 2:**
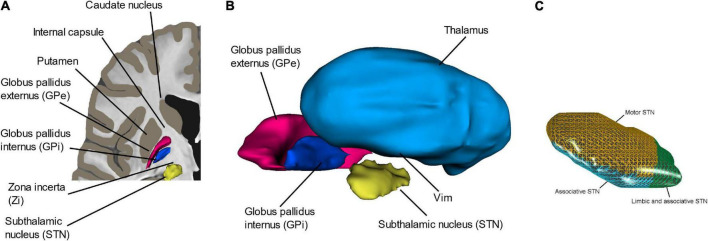
**(A,B)** The most common deep brain structures (VIM, Zi, GPi, and STN) used for DBS implantation in movement disorder. **(C)** An enlargement of the subthalamic nucleus (STN) and its motor, limbic, and associative/limbic part. Panel **(C)** with permission from Åström (2011).

The brain target region for implantation is carefully chosen based on clinical evaluation of the patient’s symptoms prior to surgery. Depending on the symptoms the implantation is done bi- or unilaterally. For movement disorders, the targets (Figures 2A,B) are in the basal ganglia, including the STN, the globus pallidus internus (GPi), the motor thalamus, and the subthalamic area. The brain targets are generally small. As an example, the STN, which is elliptic disk shaped (Figure 2C), has a volume of around 230–250 mm^3^ due to having a length of about 10 mm and a width and thickness around 5 mm (Hardman et al., 2002). Different areas of the STN can be stimulated. An extensive review of the literature of STN-DBS in PD shows support for an optimal stimulation area (“sweet” spot) without side effect located in the superior-lateral STN extending to the adjacent white matter between the thalamus and subthalamic nucleus (de Roquemaurel et al., 2021). For treatment of tremor as the only symptom, the VIM of the thalamus or caudal zona incerta (cZi) in the posterior subthalamic area (PSA) are commonly used targets (Blomstedt et al., 2010; Nowacki et al., 2018). Both VIM and cZi are located along the dentato-rubro-thalamic tract (DRT) (Coenen et al., 2014). For rigidity and involuntary muscle contractions caused by dystonia and L-DOPA-induced dyskinesia, the posteroventral GPi is stimulated. This region is passaged by the pallidothalamic tracts as explored by multifiber tractography (Pujol et al., 2016). Brain targets for psychiatric DBS are more complicated to determine as these disorders often involve a spectrum of symptoms and thus need longer evaluation time after DBS, sometimes months. This should be compared to stimulation for essential tremor in VIM or cZi where the effect of stimulation is immediate. Exploring the best fit for severe psychiatric indications is presently a topic of intensive research (Sullivan et al., 2021). As an example, up to 10 target regions have been suggested for the Gilles de la Tourette syndrome (GTS) (Visser-Vandewalle et al., 2006; Ackermans et al., 2013) where the limbic GPi is one (Akbarian-Tefaghi et al., 2017). Several targets have also been proposed and evaluated for OCD (Wu et al., 2021). Recently, the bed nucleus of stria terminalis (BNTS) and the anteromedial limbic STN were suggested as targets for DBS in OCD (Naesstrom et al., 2021). Sullivan et al. (2021) proposed widening the anatomical perspective with focus on brain targets to also include cognitive networks in the search for further understanding of DBS in psychiatric disorders.

A challenge in DBS surgery is that most of the brain structures that are aimed at are only slightly larger than the DBS lead itself. These small margins are important reasons to why the implantation procedure together with the stimulation parameter settings and lead design are of utmost importance for optimizing the stimulation outcome and to minimize side effects such as paresthesia, dysarthria, muscle, or vision affections. Furthermore, since the variations in brain tissue’s electrical conductivity can alter the stimulation field directions, not only anatomical but also physiological aspects must be considered during DBS programming and set up computer simulations of DBS. For biomedical engineers working in the DBS field, knowledge of the patient flow and techniques for planning and performing DBS surgery help in designing support tools. An update of our previous detailed presentation (Hemm and Wårdell, 2010) of these is given below.

## Deep Brain Stimulation Procedure: Preoperative Planning, Surgical Implantation, and Postoperative Follow Up

**FIGURE 3:**
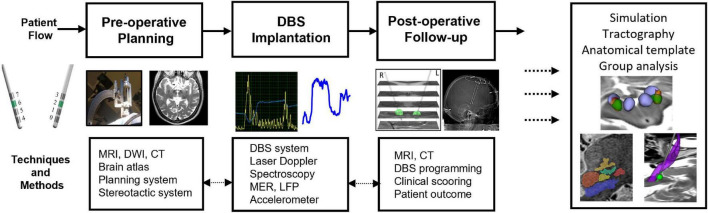
Techniques and methods for pre-operative planning, stereotactic DBS-implantation, and postoperative follow-up. Images and DBS parameters are used to set-up patient-specific simulations and tractography and to do group analysis and build deep brain stimulation atlases.

**FIGURE 4:**
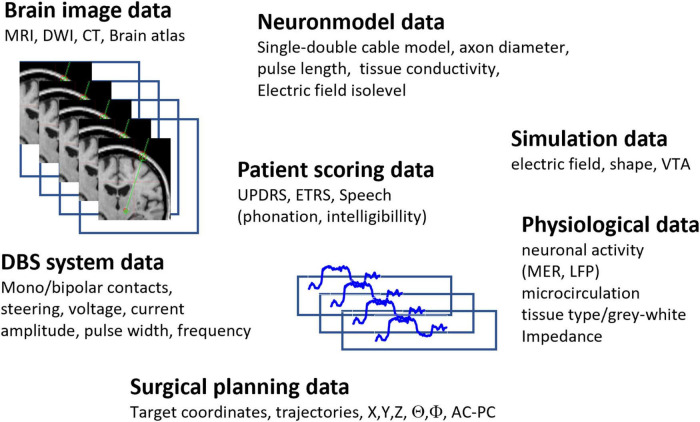
Example of multidimensional large-scale data generated during DBS planning, implantation and postoperative follow up. The data can differ between clinics.

The way in which DBS surgery is performed differs between clinics. In general, the procedure is split into preoperative planning, surgical implantation, and postoperative follow-up. The general patient flow and techniques used through these steps is illustrated in Figure 3, and a summary of parameters generated during these sessions can be seen in Figure 4.

### Preoperative Planning and Imaging

The preoperative planning starts by securely fixating a stereotactic frame, e.g., Leksell Stereotactic System (Elekta Instruments AB, Sweden) (Lunsford et al., 2009) to the patient’s skull. A magnetic resonance (MR) or computer tomography (CT) scan with the stereotactic frame and an indicator box attached to the frame is the next step. During the scanning, the stereotactic system creates landmarks (fiducials) in the preoperative images. These images are used for calculating the target coordinates and planning of the trajectory, and as reference for transforming the image coordinates to the stereotactic system coordinates set during surgery. Tailored MRI protocols have been developed for various DBS targets, and these protocols can vary between clinics (Zrinzo, 2010; Boutet et al., 2019; Krauss et al., 2021; Xiao et al., 2021). Most MR imaging sequences provide visualization of the brain structure aimed at which allows for direct targeting without reliance on brain atlas coordinates. For example, proton density sequence is preferred for visualization of the GPi. MR scans with T2-weighted setting using a long repetition time help enhance the iron-rich STN (Johansson et al., 2019). Another example is the white matter attenuated inversion recovery (WAIR) sequence developed at Clermont Ferrand University Hospital (Zerroug et al., 2016). It has been applied in a large series of patients for anatomical MRI mapping of pallidal, subthalamic, and ventral thalamic regions. Fast grey matter acquisition T1 inversion recovery (FGATIR) sequences have been proposed for subcortical structures such as the GPi (Sudhyadhom et al., 2009) and the anterior nucleus of the thalamus (Grewal et al., 2018). The imaging also often adds T1-weighted information with Gadolinium contrast for visualization of blood vessels. When the target is not, or poorly, visible in the MRI, indirect targeting is used. This means that internal landmarks, such as the anterior and posterior commissure (AC-PC) together with traditional anatomical atlases created from dissected brains superimposed on the MRI, are used.

The planning of the entry point, trajectory, and target is done on commercial software systems, e.g., Stealth Station (Medtronic Inc., Minneapolis, MN, United States) or iPlan (BrainLab AG, Munich, Germany). The trajectory and target positions are transformed to frame coordinates used during settings of the position in the operating room. A major distinction among surgical procedures is if the patient is asleep or not. Traditionally most surgeries have been done with the patient awake, as this allows for intraoperative testing of the stimulation outcome, especially for movement disorders. Along with better imaging techniques and planning tools and for the comfort of the patients, more centers switch to general anesthesia during surgery, a method used over 20 years at Montpellier University Hospital (Coubes et al., 2002) and the past 10 years at Linköping University Hospital, the DBS Unit in Umeå, and at the National Hospital in London.

### Surgical Implantation and Intraoperative Measurements

**FIGURE 5:**
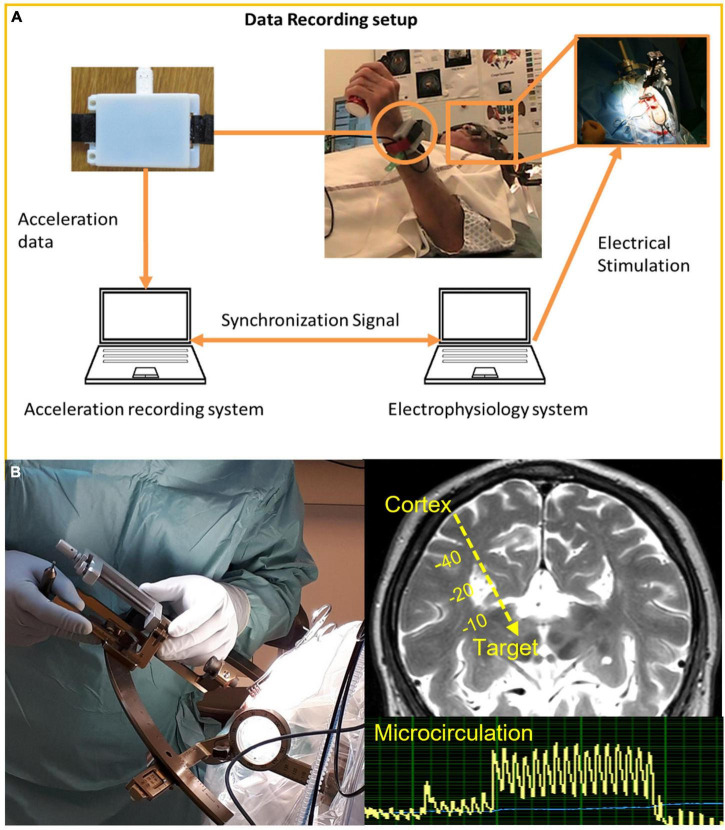
**(A)** Quantitative symptom evaluation using wrist accelerometers as response to intraoperative test stimulation during DBS implantation in awake patient. Reprinted with permission from Springer Nature: Springer, Shah et al. (2017). **(B)** Intraoperative stereotactic laser Doppler flowmetry measurement of microcirculation along planned trajectory in relation to DBS implantation in asleep patients.

During surgery, a small burr hole (about 14 mm in diameter) is created for insertion of the lead at the planned entry point. Immediately after opening of the dura, Tisseel glue (Baxter Medical AB, Sweden) is applied to avoid cerebrospinal fluid (CSF) leak and air entry and can thus help reduce the brain shift (Göransson et al., 2021). During surgery, various intraoperative techniques can help guide the surgeon to the pre-planned target. Electrophysiological methods, such as LFP and microelectrode recording (MER), are often combined with intraoperative stimulation tests. Wrist accelerometer recording of stimulation response during surgery for search of optimal target position in relation to intraoperative VIM implantations for tremor and rigidity has been described (Shah et al., 2017; Shah et al., 2020) (Figure 5A). Impedance measurements (Zrinzo and Hariz, 2008) is another intraoperative guidance methods, but with rather low resolution (Johansson et al., 2009) when compared to optical techniques such as diffuse reflectance spectroscopy (DRS) (Giller et al., 2003; Antonsson et al., 2008) and laser Doppler flowmetry (LDF) (Wårdell et al., 2013b). With forward looking optical probes adapted for the stereotactic system, both the gray-white tissue variations along the trajectory and the microvascular blood flow can be recorded intraoperatively with LDF before the tissue is even touched (Figure 5B) (Wårdell et al., 2016). A safety analysis of close to 3,000 anatomical measurements along DBS trajectories showed elevated microvascular blood flow at 7.9% of the sites (Zsigmond et al., 2017). In addition, more than five times higher blood flow was found in 2.2% of the anatomical spots, a number closely related to documented hemorrhage incidents when using MER (Tonge et al., 2015). LDF alone (Zsigmond et al., 2017) or in combination with MER and stimulation features in one probe (Wårdell et al., 2019) has a potential to identify high-risk regions (“vessel alarm”) during DBS implantations in a similar manner as fluorescence and LDF measurements during stereotactic brain tumor biopsies (Richter et al., 2021).

Many centers perform intraoperative position control of the electrode and trajectory with fluoroscopy, i.e., intraoperative 2D X-Ray. Some use an O-arm CT scan while others have a MR scanner readily accessible in the operating room. The detection of the orientation of the segmented DBS leads is a challenge. Egger suggests intraoperative 3D X-ray for identification of the lead direction (Egger et al., 2021). With the aim to intraoperatively track the DBS lead orientation in real-time and before closing the brain, the integration of magnetometers at the tip of the DBS lead is under investigation combined with the development of a magnetic tracking system adapted to the stereotactic environment in the operating room (Quirin et al., 2021). Results with a first prototype show an angle tracking precision of 2.5° ± 2.2° and are thus very encouraging (Vergne et al., 2021).

The total time for imaging, planning, and surgery vary between centers. For example, at Linköping, Umeå, and Queen Square London, the procedure is done in one session and lasts around 4–5 h, including potential optical or impedance measurements, immediate postoperative imaging (with O-arm CT or with MRI), and implantation of battery. Other centers perform the surgery as a 2-day protocol where the frame positioning and imaging are done on 1 day and the surgery the next day. If intraoperative MER is performed, the total time is increased by about 1–3 h depending on the number of trajectories needed for the recording. The most time-consuming step is the postoperative programming of the stimulation.

### Postoperative Follow Up

If not already done during surgery, a postoperative control of the final electrode position and the absence of hemorrhage is done by CT or MRI (Chabardes et al., 2015). Fusion of the pre-and postoperative images is part of a quality control as it makes a comparison between the planned and the final electrode position possible. These images are also used as input to the brain model when setting up patient-specific simulations around the active stimulation contact (see Section “Patient-Specific Modeling, Simulation, and Visualization in Deep Brain Stimulation”).

The patient follow-up consists of regular consultations. DBS parameters must be individually adapted and the final active contact, pulse width, frequency, and voltage/current fine-tuned and programed. This is done in relation to evaluation of symptom reduction and potential side effects. Scoring systems which vary depending on symptom and disorder are used. Motor symptoms in PD, for instance, are evaluated by the Unified Parkinson’s Disorder Rating Scale (UPDRS) part III. Sometimes, speech analysis is added (Tripoliti et al., 2008). Tremor is evaluated with the Essential Tremor Rating Scale (ETRS). In principle, all disorders/symptoms have their own scoring protocol.

## Patient-Specific Modeling, Simulation, and Visualization in Deep Brain Stimulation

Simulation methods of the volume around a DBS lead [also denoted as volume of tissue activated (VTA) or volume of neural activation (VNA)] have been proposed by several groups (McIntyre et al., 2004; Hemm et al., 2005; Butson et al., 2007; Åström et al., 2009; Madler and Coenen, 2012; Schmidt and van Rienen, 2012). A key issue in setting up simulations and making them patient-specific is to use as many realistic parameters as possible. Parameters of importance to consider are lead design and active contact, stimulation mode and parameter settings, and the brain tissues’ properties at the actual anatomical stimulation site. The DBS system parameters are easily achieved from respective patients’ settings, but brain tissue properties, such as electrical conductivity (σ), rate of anisotropy, peri-electrode space (PES), neuronal density, and axon size and direction, are difficult to acquire and often dependent on indirect measures. In addition, the DBS lead must be placed at that same anatomical site of the model as in the patient simulated to mimic the actual clinical situation as much as possible.

### Physical Properties

The electrical conductivity is commonly estimated from indirect measures which have been transformed into tabulated values (Gabriel et al., 1996; Audreccetti et al., 2005) or indirectly *via* diffusion weighted imaging (DWI) (Tuch et al., 2001; Åström et al., 2012; Schmidt and van Rienen, 2012). MRI protocols for brain tissue electrical property estimation have been suggested but are not available on a routine basis (Chauhan et al., 2018; Mandija et al., 2021). The electrical tissue conductivity varies depending on brain tissue and is highest in CSF. Based on Gabriel et al. (1996), conductivity values at 60 μs and 130 Hz are 0.12 S/m (gray matter), 0.07 S/m (white matter), 0.7 S/m (blood), and 2.0 S/m (CSF). For gray and white matter, σ is slightly dependent on frequency and pulse width, but there are uncertainties in the literature of the conductivity values and its frequency dependency for different tissues (Schmidt et al., 2013; Chauhan et al., 2018). Rate of anisotropy can be important to consider in white matter where the conductivity can vary up to ten times depending on the fiber direction (Schmidt and van Rienen, 2012; Nordin et al., 2020). The thickness of the PES surrounding the DBS lead changes over time from extracellular fluid in the acute stage to fibrosis in the chronic stimulation situation (Yousif et al., 2008). It can be included in the model as a thin layer around the lead with a σ depending on if the simulation should mimic a time point directly after implantation or the chronic DBS phase (Alonso et al., 2015; Alonso, 2018).

The actual axonal diameter, density, and direction in the brain target region is not possible to know and is instead based on anatomical and histology investigations of brain slices. Studies performed in the last decade show the presence of axon thickness below 0.5 μm in the deep brain (Mathai et al., 2013; Liewald et al., 2014), which is smaller than previously assumed. Neuron models are used to describe and mimic the transmission of nerve signals along an axon, and both single cable and double cable models exists. While single cable models are valid for a continuous range of small axon diameters calculated for each iteration, fixed defined axon diameters are applied in double cable models. The first finite element method (FEM) model for DBS (McIntyre et al., 2002; McIntyre et al., 2004) was combined with a double cable model in NEURON^®^ for the simulation of VTA and further developed to be patient-specific (Butson et al., 2007; Chaturvedi et al., 2010; Gunalan et al., 2017). In the first version, it was implemented with a fixed model-dependent axonal size of 5.7 μm. The neuron modeling technique was later extended for axons with 2 and 3 μm diameters by Sotiropoulos and Steinmetz (2007) and, thereafter, also for thicker axon diameters, i.e., 7.3, 8.7, and 10 μm (Schmidt and van Rienen, 2018; Latorre and Wårdell, 2019). The Linköping University (LiU) concept for patient-specific DBS simulations (Åström et al., 2009) is linked to a single cable model developed in MATLAB^®^ (MathWorks^®^ Inc., United States) by Hubert Martens, and valid for any axon diameter within the range of 1.5–10 μm. Meanwhile, a full description of the single cable model has been presented by Åström et al. (2015). For a systematic comparison between the single and double cable axon models for parameters typically used in DBS applications, the reader is referred to Latorre and Wårdell (2019).

### Linköping University Brain Modeling and Finite Element Method Simulation

**FIGURE 6:**
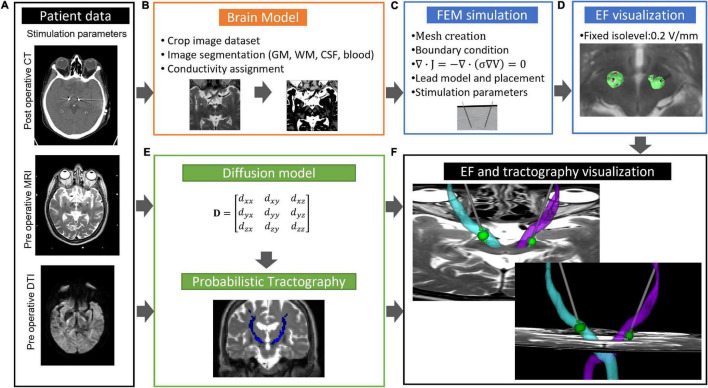
Overview of workflow for patient-specific EF simulation and tractography. **(A)** MRI and stimulation parameters; **(B)** creation of brain model; **(C)** set up of FEM simulation; **(D)** visualization of electric field superimposed on preoperative MRI; **(E)** diffusion model for probabilistic tractography; and **(F)** visualization of electric field, tractography superimposed on preoperative MRI. GW, gray matter; WM, white matter; CSF, cerebral spinal fluid.

An overview of the LiU approach (Åström et al., 2009; Åström, 2011; Wårdell et al., 2011; Alonso et al., 2016; Johansson et al., 2019; Nordin et al., 2019) for patient-specific DBS simulations is presented in Figure 6. The workflow starts by building an electrical conductivity brain model from the patients undergoing preoperative T1, T2, or PD MRI (Figures 6A,B). Each voxel in the MRI is replaced with the corresponding σ to create a heterogeneous brain model. Next, the tissue is classified into gray and white matter and blood and CSF and assigned their corresponding σ based on the frequency and pulse width settings (Gabriel et al., 1996; Audreccetti et al., 2005). A software-compensation is done for variations in DBS stimulation frequency and pulse width (Wårdell et al., 2013a). To minimize the errors, a linear interpolation is done between neighboring voxels (Åström et al., 2009). These steps result in a 3D conductivity volume (brain model or volume conductor model) which is used as in-data for the FEM-simulations (Figures 6C,D). To shorten the FEM calculation time, a user selected region of interest (ROI, typically 100 mm × 100 mm × 100 mm) including the target and the closest brain structures is selected from the brain model before creating the mesh and defining the boundary conditions. This model assumes a quasistatic case and thereby neglect the capacitive effect. While the capacitive effect has an impact on the tissue-voltage response, it has limited impact on thresholding approaches (Butenko and van Rienen, 2021) where an isolevel (see Section “Visualization”) is set to estimate tissue activation. By doing this approximation, the computational cost is significantly reduced.

The patient-specific positioning of the lead model in the brain model is most important (Athawale et al., 2019). The use of the artifacts from the postop CT or MR images as suggested by us (Hemm et al., 2005; Åström et al., 2009) is now a common method for lead placement (Horn and Kuhn, 2015; Egger et al., 2021). To select two positions along the lead artifact from the planning system in the co-registered postoperative image is also a possibility for placement of the lead in the model (Johansson et al., 2019). Any method requires an image inspection of a person skilled in DBS surgery to verify the correct placement of the lead. When using steering leads, the identification of the stimulation direction is essential for setting up the models. As the DBS lead can rotate after its implantation, it is important to know its direction in relation to the anatomy. Algorithms for determining the lead direction after implantation have been suggested based on stereotactic CT images (Sitz et al., 2017; Hellerbach et al., 2018) and 3D fluoroscopy (Egger et al., 2021). Once the predesigned DBS lead is positioned in the brain model and boundary conditions are applied, the equation of continuity for steady currents is used for calculation of the electrical potential in the vicinity of the active contact at the lead (Figure 6C). This calculation is done within a few minutes on a standard laptop with COMSOL Multiphysics (COMSOL Multiphysics, AB Sweden). The run time is, however, always dependent on the total number of mesh elements and their size within the preselected ROI which is used for creation of the brain model (Åström et al., 2009; Alonso et al., 2016; Hemm et al., 2016; Nordin et al., 2019). Convergence test should be done to find a trade-off between these parameters. Our group has used COMSOL Multiphysics (version 3.0–5.6) for DBS-FEM modeling and simulation since 2004 and are continuously updating and refining the methodology in parallel to new COMSOL-software versions and DBS technology developments. Recently, the workflow (Figure 6) was updated for combined patient-specific visualization of probabilistic tractography, simulated EF based on anisotropic σ, and MRI (Nordin et al., 2019).

### Visualization

For visualization, the LiU concept uses the EF (Figure 6D), i.e., the potential’s first derivative, superimposed on the patients’ own preoperative MRI (Åström et al., 2009). Previous investigations have shown that the EF approximates the activation distance for a specific axon diameter, pulse width, and stimulation amplitude without the need to couple the single axon model to each specific FEM solution (Åström et al., 2015). Using this approach, a typical isolevel of 0.2 V/mm refers to an axon size of approximately 3 μm at 60 μs. The 0.2 V/mm isolevel was first suggested by Hemm et al. (2005) who correlated the EF to the clinical effect and the absence of side effects. How the isolevel, axon diameter, and pulse width interplays can be found in Åström et al. (2015) and has also been further explored by us (Alonso et al., 2016; Latorre and Wårdell, 2019). Major advantages with EF are that relative comparisons between simulations can be done when a fixed isolevel is chosen, and that the EF can be displayed superimposed on the patients preoperative MRI using the same scale, i.e., V/mm (Figure 6D). This makes direct anatomical inclusion possible in the visualization and opens for patient group analysis (see Section “Deep Brain Anatomical Templates and Group Analysis”). Several other groups are now following this visualization approach (Coenen et al., 2014; Akbarian-Tefaghi et al., 2017; Horn et al., 2019).

Other visualization methods for presentation of simulation results are sometimes also applied. Particularly, the potential alone or as the potentials second derivative (V/mm^2^), i.e., the activating function (Rattay, 1986), as commonly used by McIntyre and colleagues. When using DWI atlas-based information to set the conductivity in the models, the activating function is a useful approach (Butson et al., 2007; Chaturvedi et al., 2010). The Butson group has continued to improve the DBS-FEM simulation methodology (Anderson et al., 2018) and also recently suggested a computer model taking the 3D directions of the activation function into account (Duffley et al., 2019). Comparisons between the visualization of the EF and activating function methods have been previously presented by Åström et al. (2009, 2012).

### Open Access and Commercial Simulation Software

**FIGURE 7:**
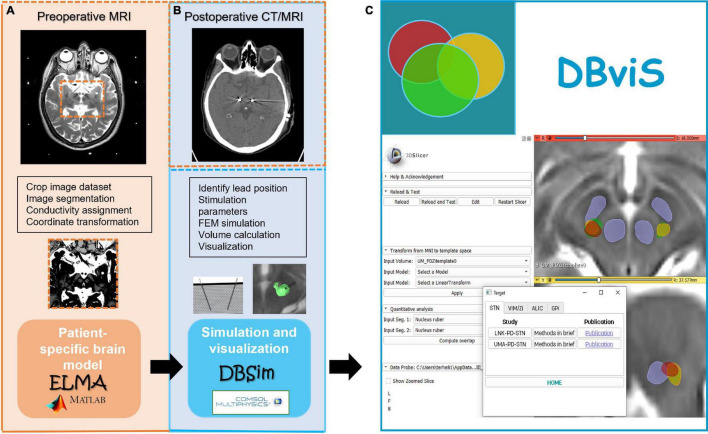
**(A)** Brain model creation and **(B)** patient-specific electric field simulation applications (APPs), and their link to the modeling and simulation software. The concept in collecting, presenting, and comparing DBS simulation studies using the DBviS APP is shown in **(C)**.

The LiU patient-specific DBS modeling and simulation method is available as non-commercial open access applications (APPs)^1^. It consists of two APPs, ELMA (Figure 7A) and DBSim (Figure 7B), which originate from the patient-specific modeling, simulation, and visualization concept. Both APPs are available for PC, Mac, and UNIX environment and are controlled through a graphical user interface which allows for setting input parameters. ELMA (Wårdell et al., 2011; Johansson et al., 2019) is programed in MATLAB^®^ and used for building the conductivity brain model and further used as input to DBSim, the APP where the actual FEM simulations and visualizations are done. The DBSim is generated using COMSOL Multiphysics and COMSOL Complier™ (COMSOL AB, Sweden) where the boundary condition, mesh size, and governing equations are pre-programed based on our previous experiences (Åström et al., 2009, 2015; Alonso et al., 2016; Hemm et al., 2016). By using COMSOL Compiler™, DBSim becomes open access and free for the users as they do not need to purchase a COMSOL License. In DBSim, pre-programed lead designs, mono-, and bipolar stimulation can be chosen with and without PES, along with user settings of voltage and current stimulation amplitudes. The electric field is visualized as superimposed on the patient’s own preoperative MRI. The user can as well change the isolevel based on the expected axon diameter in the target area and thus repeatedly update the visualization with different settings. As an option, the volume within the chosen isosurface is calculated. Simulation results can also be exported to other visualization and analysis softwares. With DBSim, a FEM simulation is done within a few minutes.

Also, other researcher groups have developed pipelines for DBS simulations. The Rostock group (Butenko et al., 2020) recently suggested an open-source simulation platform for DBS (OSS-DBS). They built a comprehensive automated modeling pipeline from their own software and verified it against COMSOL Multiphysics. Anderson et al. (2018) extended their simulation method and made it available *via* SCIRun Software. They also did a systematic comparison of the simulated VTA for monopolar stimulation and found a good agreement between methods (Duffley et al., 2019). LeadDBS is yet another research tool for DBS simulations and it is incorporated as a module in a Matlab Toolbox (Horn et al., 2019). Their VTA determination is based on the discrimination of gray and white matter by fitting an anatomical atlas on the patient’s images, which doesn’t take patient-specific cysts and blood vessels into account [(Horn et al., 2019), supplementary material]. According to the authors, the VTA calculation is a mixture between the LiU (Åström et al., 2009) and McIntyre’s (McIntyre et al., 2004) approaches and implemented with the Fieldtrip-SimBio (Horn et al., 2017b). The user can also choose among several other empirical methods (Kuncel et al., 2008; Madler and Coenen, 2012; Dembek et al., 2017). Pre-simulation VTA models form the base for visualization of the EF for different DBS leads. Recently, OSS-DBS (Butenko et al., 2020) was also implemented in LeadDBS.

Today, there are also several commercial DBS simulation softwares available in the market. Boston Scientific simulation package (Guide™ DBS System) is a further development from McIntyre and Butson’s Ciceron reference volume methodology (Miocinovic et al., 2007). SureTune™ (Medtronic Inc., Minneapolis, MN, United States) originates from the LiU approach (Åström et al., 2009), but without the option of taking various electrical conductivities into account when building the brain model. It was further developed within the FP7 EU project IMPACT coordinated by Sapien Steering Brain Stimulation in The Netherlands before the company was acquired by Medtronic Inc., Minneapolis, MN, United States. For voltage simulations, variation in conductivity has limited impact, but for the current mode, the conductivity is of high importance as shown by Alonso et al. (2021). A systematic comparison between the Suretune3 and the ELMA/DBSim concepts confirm this finding (Johansson and Zsigmond, 2021).

### Clinical Applications

Over the last decade, the use of DBS simulations has increased to more clinical applications. A few examples are given below from our own experience. In addition, studies were performed together with clinical DBS researchers at Umeå DBS Unit and Department of Neurosurgery in Linköping, both in Sweden, and the Department of Neurosurgery at Clermont Ferrand University Hospital. France, and Functional Neurosurgery at Institute of Neurology, London University College. More examples, also from other groups, are given where the VTA and tractography are combined (see Section “Tractography in Deep Brain Stimulation”), and examples of VTA probabilistic mapping are discussed under Section “Deep Brain Anatomical Templates and Group Analysis.” From a methodology and technical aspect, we have used the FEM simulations for investigation of lead designs influence on the tissue properties. In a paper by Alonso et al. (2015) it became obvious how the extended tip of a 6148 lead (Figure 1C) caused stimulation in unexpected regions. This particular lead is not available on the market anymore. A simulation comparison between intraoperative test stimulations using MER and DBS electrodes inducing the best clinical outcome showed a deviation between the two VTAs (Alonso et al., 2018). The study highlights the differences in the generated EF for the two electrode types. Different lead designs (contact size and area), stimulation set-up (grounding method and stimulation mode), and the presence of conductive material in the vicinity of the stimulating contact (guide tubes, parallel MER electrodes) influence the distribution and might, in consequence, be responsible for different clinical results. Alonso et al. (2021) also used patient-specific EF simulations to investigate Virchow-Robin space, i.e., CSF-filled cystic cavities in the STN region, and found that these can alter the electric field. We have also combined STN DBS simulations with patient-specific investigations of the volume of influence around microdialysis catheters positioned in the GPi and putamen (Diczfalusy et al., 2011, 2012). This shows the possibilities to expand the fundamental modeling methodology to other research investigations and fully use the options in COMSOL Multiphysics. Simulations with the LiU approach have also been applied for introducing the concept to identify the optimal implant position based on intraoperative test stimulations and the induced improvement of tremor. Quantitative measures of wrist movements with accelerometers (Figure 5A) were automatically linked to intraoperative test stimulations to find a threshold with the clinical effect (Hemm et al., 2016). Patient-specific electric field studies have been applied for both movement disorders such as PD and ET (Åström et al., 2010; Alonso et al., 2018; Göransson et al., 2021; Stenmark Persson et al., 2021) and in relation to psychiatric indication such as GTS (Wårdell et al., 2015; Akbarian-Tefaghi et al., 2017) and OCD (Naesstrom et al., 2021).

## Tractography in Deep Brain Stimulation

### Diffusion Weighted Imaging and Estimation of Fiber Tracks

An important aspect to consider in DBS surgery is the location of white matter fibers in the vicinity to the target region and other connected brain areas of importance. These can be investigated with tractography, but requires DWI, i.e., MR signals sensitive to the motion of the water molecules. As for all measurement systems, a key issue is the quality of data collection which, for tractography, originates from pre-programed MR-scanner-dependent DWI protocols. Parameters including number of gradients and *b*-values, repetition- and echo time of pulse sequence, and voxel size need to be fine tuned to optimize the signal quality for a specific MR camera. Normally, a longer MR scan time gives a higher signal-to-noise ratio and thus improves quality of the diffusion data for the tractography calculations. For a patient situation, however, it will always be a trade-off between scanning time, i.e., what can be allowed in a patient with tremor or other movement symptoms, and the image quality. The mathematics behind the calculations of the anatomical white matter tracts also differ. The major fiber tracking methods used in DBS are either deterministic or probabilistic or can even be a mix between these two. Also, the seeding points are of high importance. Pujol et al. (2015) at Lab of Mathematics, Harvard Medical School, did a systematic investigation of variation in seeding points, diffusion models, and fiber tracking methods of the pyramidal tract and showed that in principle any trajectory can be extracted and visualized if care is not considered in every step of the workflow. This obstacle is due to the inherence of the methods base in the statistical calculations, choice of seeding points, visualization method, and MR data collection. In short, the white matter fibers are difficult to reconstruct in a realistic and reliable way, and care must be taken by every single step along the workflow for the best and most realistic result.

### Tractography and Deep Brain Stimulation

The Linköping group has developed, together with the above cited Lab of Mathematics, a workflow for patient-specific probabilistic calculations of detailed white matter tracts as the DRT (Figures 6A,E,F). The DWI protocol takes approximately 8 min to run, which is a realistic scan time for patients with movement disorders. Further details regarding the protocol and workflow can be found in Nordin et al. (2019). A full implementation of this comprehensive workflow was used for the DRT on four patients with essential tremor implanted in the cZi. The precentral gyrus was used as seeding region, while the superior cerebellar peduncle and dentate nucleus were used as waypoints. The white matter crossing fibers were combined with patient-specific EF simulations, and the result was visualized together with the respective individuals anatomical MRI using 3DSlicer developed at Harvard (Fedorov et al., 2012). An example of crossing DRT fibers is shown in Figure 6F. It is clearly seen that the simulated EF overlaps with the DRT. We are now further improving the DWI procedure and transferring an updated version of the workflow to other brain regions relevant for DBS and other neurosurgical applications. As a next step, the open access APPs ELMA and DBSim will be updated with possibility to visualize reconstructed tracts and simulate with anisotropic conductivity.

Tractography has also been suggested as a tool for supporting surgical planning in DBS by Coenen et al. (2011). They hypothesized that the DRT-white matter tract could be useful for DBS surgery planning, especially as three of the most common targets, VIM, Zi, and STN, are along the DRT (Coenen et al., 2014). Their first studies used single tensor deterministic tractography as implemented in StealthViz (Medtronic Inc., Minneapolis, MN, United States). Thus, crossing, kissing, and branching fibers were not possible to visualize. Recently, they scrutinized several DWI scanning protocols and calculation methods for commercial tools and still found missing crossing fibers and variations in reproducibility when investigating the DRT (Coenen et al., 2021). By combining streamline calculations with machine learning, tractography, as a support tool in planning of certain targets, can find larger reliability and use (Coenen et al., 2019).

### Cortical Connectivity and Deep Brain Stimulation

Other groups have mapped out the cortical fingerprint from STN stimulations. Akram et al. (2017) developed a comprehensive workflow for probabilistic tractography from patients’ DWI and combined tractography with patient screening and SureTune™ simulations. The Montreal Neurological Institute (MNI) brain (Grabner et al., 2006) was used as standard space for visualization of the 20-patient group averaged tracts and VTAs. As previously described, the use of SureTune™, which is a simplified modeling and simulation technique, does not fully take the local tissue conductivity into account. Also Andrade and co-workers used probabilistic tractography and MNI together for retrospective exploring the tracts and VTAs through LeadDBS for GTS patients implanted in the thalamus (Andrade et al., 2020). They expanded their studies to GPi thus with sham VTAs (Heiden et al., 2021). The use of tractography in psychiatric DBS and GTS is increasing, and new investigations continue to give their input in the search for the best implantation spot (Johnson et al., 2020).

Other DBS studies have presented pipelines and studies of both cortical brain structural connectivity and functional connectivity mapping for STN-DBS with the aim to predict the outcome in larger cohorts (Horn et al., 2017b). It is, however, important to point out the difference between the two methods, whereas structural connectivity (tractography) is based on DWI as described above, functional connectivity originates from resting-state functional MRI and thus measures correlations from spontaneous variations in the blood oxygenation (BOLD) signal since no specific task is performed (Horn et al., 2021). Wang et al. (2021) did a comparative study between patient-specific and normative structural connectivity and suggests that given an optimized DWI protocol, individualized structural connectivity would have a slightly better potential to estimate clinical outcome following STN.

## Deep Brain Anatomical Templates and Group Analysis

Probabilistic mapping of the electric field in larger cohorts is an important aspect to consider when evaluating the stimulation field efficacy of a specific target and its relation to symptom reductions and potential side effects. A major difficulty for data analysis and interpretation and their optimal use for planning of the implantation and chronic stimulation is the individual variability in brain anatomy. The analysis and use of data on a group level can be used as support. The concept consists of transforming each patient’s brain images (MR/CT) to a common reference (anatomical template) and to project other information, such as final implantation position (delineated structures, contact positions, and stimulation efficacy), into the template to analyze the relation between anatomy, stimulation, and symptomatic and adverse effects.

### Anatomical Template Generation

Nowinski et al. (2005) was first to suggest normalizing anatomies and best contact locations of a larger cohort implanted in the STN. These were achieved in relation to the Schaltenbrand-Wahren atlas (Schaltenbrand and Bailey, 1959) by using affine transformations. Further extensive work has concentrated on optimizing the tools (Smith et al., 2004; Ashburner, 2007; Avants et al., 2011) and methods (Grabner et al., 2006) to create anatomical templates. This has resulted in the creation of different anatomical references built of anatomical images from many individuals. Prime examples are the anatomical templates from the MNI, such as the often-used ICBM MNI 2009b non-linear asymmetric template (Fonov et al., 2011). The shortcoming of using a template built with a population different from the group under study is that the template may introduce anatomical bias depending on the type of population selected (Lancaster et al., 2007; Avants et al., 2010) and the image data available. This can lead to a lack of details of the small deep brain structures which are of high importance for DBS procedures (Ou et al., 2014). For that reason, several studies have been based on group-specific templates from patients undergoing DBS (Åström et al., 2018; Johnson et al., 2019).

**FIGURE 8:**
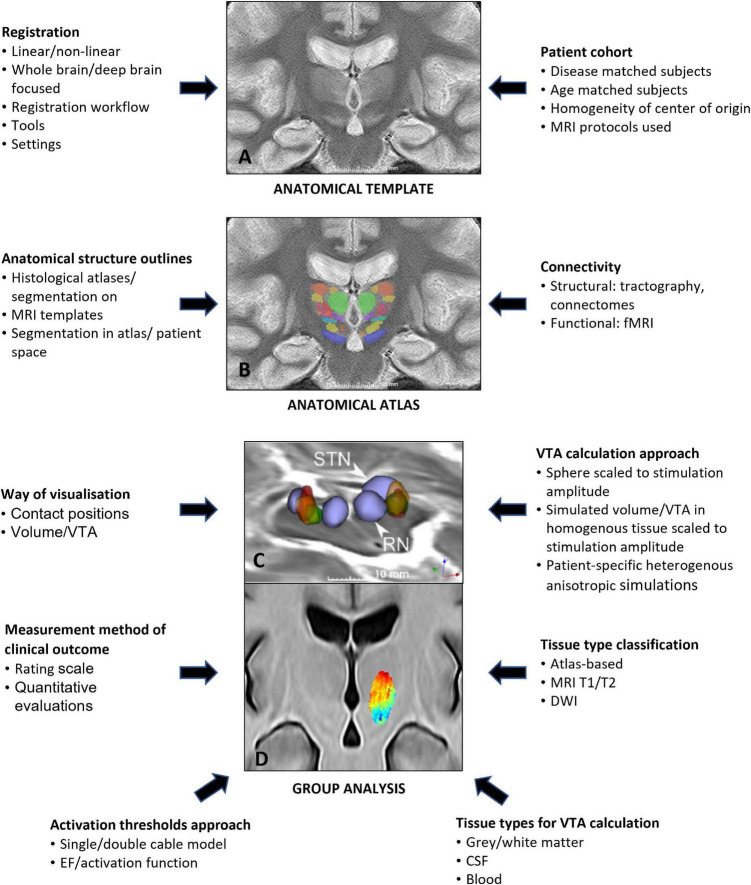
Example of a deep brain anatomical template alone **(A)**, an anatomical atlas with structural information projected on the anatomical template **(B)** and stimulation atlases with stimulation maps from patient cohorts projected on the anatomical template for group analysis **(C,D)**. Parameters that can influence the different steps are noted on the left and right sides.

The present research in our consortium follows a similar approach to create group-specific anatomical references using multiple iterations of state-of-the-art non-linear image registration (Figure 8). In our first study which included 15 patients with PD implanted in cZi (Stenmark Persson et al., 2021), a group average brain template was built from pre-operative T1 MRI only, and non-linearly transformed with Advanced Normalization Tools (ANT) (Figure 8C). Further developments use an iterative mixed-modalities approach with finely tuned settings (Vogel et al., 2021) to preserve details in the deep brain provided by both T1 and the WAIR images of 19 patients with PD and ET (Vogel et al., 2020, 2021) (Figure 8A). The same approach was applied to pre-operative T1 and T2 MRI scans to create a template from a larger cohort (*n* = 71) of patients with ET with implants in the cZi (Figure 8D) (Nordin et al., 2021b).

### Anatomical Atlas

The anatomical templates represent the basis for a group analysis, i.e., for combining several sources of information and more specifically for analyzing the correlation and the overlap between anatomy and electrical stimulation for a whole patient group. Sources of normalized anatomical information may be of different levels of details. Some studies used histology atlases, such as the Schaltenbrand-Wahren atlas (Nowinski et al., 2005) and Morels atlas (Åström et al., 2010; Fytagoridis et al., 2013), or atlases more specific to the basal ganglia, such as the one from Mai et al. (2015), Dembek et al. (2017), and Horn et al. (2017a). Another approach is integrating segmentations from histology atlases into a probabilistic MRI atlas (Ewert et al., 2018) to obtain the delineations of the anatomical structures. Both types of atlases bring in the known shortcomings of histological atlases (Vayssiere et al., 2002). Our approach to integrate additional anatomical information is based on manual delineation. This was either done by segmenting STN and red nucleus in the anatomical group template (Stenmark Persson et al., 2021) (Figure 8C) or by using patient-specific manual delineation of 58 deep anatomical structures on T1/WAIR datasets from a single expert, a method developed by Lemaire et al. (2010) and Lemaire (2021). These structures were then projected into the template to build an anatomical atlas (Vogel et al., 2020) (Figure 8B).

### Stimulation Maps and Atlases

To estimate the electrical effect of stimulation in a patient, several authors include the location and symptomatic effect of the contact of the electrode used for long-term stimulation (Fytagoridis et al., 2013; Lalys et al., 2013). In contrast, a more precise estimation can be done by using *in silico* simulations of single patient distribution of the EF (Åström et al., 2010, 2018; Hemm et al., 2016; Akbarian-Tefaghi et al., 2017; Naesstrom et al., 2021). Implementation of this method can also be expanded to visualize induced improvements and adverse effects in patient-specific stimulation maps (Shah et al., 2020). Stimulation maps from several patients can then be projected into the anatomical template space to set-up disease specific stimulation atlases as we have generated for patients with ET with implants in the VIM (Vogel et al., 2021) and in the cZi (Nordin et al., 2021b; Stenmark Persson et al., 2021). Examples are shown in Figures 8C,D. Methods for prediction of contact settings have also been suggested (Åström et al., 2018; Reich et al., 2019).

After normalizing/stacking the results of different EF simulations in the same space (be it single patient’s images or group template), several improvement values per voxel are available from an individual patient and/or from a patient group. This information has to be summarized, resulting in a stimulation map with one value per voxel. Different ways of summarizing have been proposed. Examples are unweighted frequency “n-map” (Elias et al., 2021), minimum, maximum (Shah et al., 2020), and weighted “mean-maps” (Nordin et al., 2021b) or “p-maps” denoting the degree of confidence for the stimulation at a voxel associated to a clinical change (Elias et al., 2021). Once the stimulation atlas is set-up including the anatomical template, some researchers project information such as coordinates of activated electrode contacts (Dergachyova et al., 2018) or connectivity profiles (Horn et al., 2017b) to new patients to test the predictability of target location.

### Parameters Influencing Group Analysis

As described above, group analysis in DBS is a multi-level problem with each element having several compartments of complexity possibly impacting the final analysis conclusions. de Roquemaurel et al. (2021) recently published a review searching for the “sweet” spot within the subthalamic area for PD. Even if their assessment criteria were subjectively chosen, their results reveal the low quantity of high-quality publications with only few papers using VTA estimation as a criterion. This underlines the need for more high-quality publications. Furthermore, it is extremely important to be aware of all the parameters impacting the result quality. Examples of parameters are given in Figure 8. This includes data used for template generation (healthy subjects/patients; whole brains/deep brain structures; used MRI sequences), the methods and tools applied for registration (linear/non-linear; registration workflows, and settings), and the origin and quality of the available anatomical information being the basis for the interpretation. Further parameters concern the way stimulation results are displayed in template space (contact positions or VTAs), the way VTAs have been defined (patient-specific or not) and tissue types for EF simulations determined (atlas-based, MRI T1/T2 or DWI derived), the number of tissue types derived (gray/white matter, CSF, and blood), the quality of the field data projected onto the patient or the stimulation atlas (image resolution or higher), and the kind and quality of the clinical data linked to these simulations (clinical scales, quantitative evaluations). The influence of the chosen approaches/parameters on the results has to be further investigated in the future in order to get a better idea of the quality of the results obtained. Our research will continue focusing on disease-specific, adapted state-of-the-art MRI-based template generation. Together with structural information from manual segmentations and patient-specific EF simulation, the approaches take into account all tissue types to provide high quality analysis.

## From “Mental Imagination” to “Intuitive Visualization”

The amount of data collected during DBS planning, surgery, and follow up (Figures 3, 4) is big and will continue to expand with the introduction of more stimulation options, imaging sequencies, measurement techniques, and evaluation protocols. It will therefore become even more difficult for the neurosurgeon and neurologist to interpret all available data in order to take a decision on the final surgical target and the stimulation parameters by “mental imagination.” Therefore, data-driven support systems which can interact with the user and visualize the necessary information in an intuitive way will be required. Examples of methods for handling different types of DBS data and examples of patient-specific simulation and visualization methods have been presented in this review. Comprehensive pipelines for combining and visualizing atlas data, simulations, and tractography in DBS have been suggested by several groups (Akram et al., 2017; Horn et al., 2019; Nordin et al., 2019). Still, none of these workflows are complete and all have, in different aspects, development possibilities. An interesting concept is the holographic interface for visualization of the deep brain and related pathways as developed by Petersen et al. (2019). In our consortium we are working on a visualization concept, DBviS (Figure 7C), (Nordin et al., 2021a) with the aim to guide clinicians and DBS researchers to find a way forward in the massive information flow. It will be available as an open access application and will be continuously updated with new studies and patient-specific DBS simulation comparative possibilities through the ELMA and DBSim APPs. DBviS is built in 3D Slicer and will be one step closer toward intuitive visualization of our DBS studies.

Compared to the traditional statistical and data analysis methods, novel approaches are necessary to explore when the amount of DBS-data increases even more. Artificial intelligence (AI) has regained interest in mining big data and are used to train networks for creating humanlike systems for precision care of neurological indications, among these are movement disorders (Patel et al., 2021). Watts et al. (2020) proposed machine learning (ML) applications in DBS with the focus on PD. They suggest DBS candidate selection, surgical targeting, and programming optimization as the most likely areas where ML can be applied. Few groups have, however, implemented the ideas with real DBS data. Some examples of applications are deep learning of fuzzy recurrence plots as early detection and candidate selection of PD signs through the interaction of keystroke time (Pham et al., 2019). For planning of DBS surgery ML was applied to build white matter tracts in major depression for the medial forebrain bundle (Coenen et al., 2019). Other groups have focused on algorithms for DBS targeting of the STN. Park et al. (2019) developed a deep learning method from MRI records and successfully evaluated it in two implantations, while Baumgarten et al. (2017) suggested a data-driven method for prediction of STN stimulation. Peralta et al. (2021) proposed a patient screening support workflow (PassFlow) for prediction of post-operative clinical outcomes in PD. This group at University of Rennes used information from their patients operated in STN, GPi, and VIM to program a multimodal ML-based workflow. It is a promising method, but more patient data will be necessary to include for training of the network to further increase the statistical performance. The Toronto group also retrospectively applied ML for their operated patients with STN-PD to classify “hot” and “cold” spots (Boutet et al., 2021). They also built a ML model to investigate if fMRI can predict stimulation settings (Boutet et al., 2019). With further development and evaluations, fMRI may evolve to a complementary method in DBS programming assistance. These examples show that ML also has a place in the DBS research but work still remains before fully developed support systems are available. With increase of patient information, the data-driven methods can be refined and find a place in future support and visualization systems. To develop such systems requires a close collaboration between neuroengineering scientists and clinicians in the DBS field.

## Discussion and Conclusion

**FIGURE 9:**
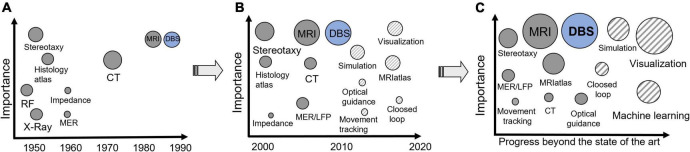
**(A)** Supporting techniques for DBS implantation and their approximate time of introduction in functional neurosurgery. **(B)** Progress during the last 20 years and state of the art. **(C)** Stipulated progress beyond the state of the art. The size of the circles shows the importance/use today. The striped circles represent novel tools.

Deep brain stimulation is a very technology-intensive domain. A high quantity of data is recorded before, during, and after the intervention. Patient-specific use of DWI for tractography and other MR sequences significantly increases the amount of data. Intraoperative data for creating closed loop systems, accelerometer measurements, and optical guiding together with brain atlases and electric field simulations are to be linked to patient records and visualized in an intuitive way. This requires smart systems that can support the clinicians in their planning, surgery, and postoperative evaluation. More than 10 years ago, we suggested (Wårdell and Hemm, 2009) that information technology was the key to improving management and visualizations of DBS data and for implementing new supporting technologies to optimize the trajectory planning and final stimulation target choice (Figures 9A,B). We also proposed that the “mental imagination” should be replaced by “intuitive visualization” (Hemm and Wårdell, 2010). Today the number of research groups and companies working on these topics have increased. Together with improved software tools and high-capacity computers, the DBS community is getting closer to user-friendly visualizing tools facilitating final target choice. It is, however, of outmost importance that users give feedback and question the methods behind the presented results. In the next decade we stipulate that deep learning, ML, and, possibly, AI together with “intuitive visualization” tools will have a high impact on improvement of the support systems (Figure 9C).

But how will the presented tools and methods improve clinical practise? The commercial systems allowing to visualize and compare the VTA for different stimulation approaches are helpful to give an idea of how stimulation look like. Nevertheless, to support neurologists in selecting the optimal stimulation parameters, more complex simulation and patient-specific approaches might be necessary. Disease-specific stimulation atlases taking into account the results of a whole patient population and providing information about “sweet” spots and adverse effect regions along with ML approaches will support parameter programming and surgical planning. Better knowledge about the optimal implant position thanks to identified areas inducing therapeutic and adverse effects from a large amount of data will hopefully result in shorter and a reduced number of programming sessions. The implementation of high-quality fiber tracking in clinical practice will add additional patient-specific information about fibers crossing the target area and responsible for certain clinical effects. Furthermore, the presented techniques can help reduce the surgical planning and intraoperative test session times and further support the transfer from awake to asleep DBS surgeries. The prerequisite to succeed this step is, again, certainly to be aware of, to question, to investigate the different available approaches, and, probably, to push their connection forward. Nearly each clinic has its own implantation strategy, imaging protocols, and different kinds of further available valuable data. An essential step would be to intensify the movement from single-center to multi-center studies and data analyses to increase patient cohorts and to combine results from different targeting approaches and data sources for setting up large data bases and disease-specific stimulation atlases. There are, however, no shortcuts to the next steps in DBS, and the basis will always be the input of data. Therefore, future systems as electric field simulations, tractography, and brain atlases should be based on as much patient-specific information as possible to provide realistic information to the end users in an accepted time perspective relevant to clinical settings.

## Author Contributions

KW initiated and was overall responsible for the study. KW and SH performed the overall tasks including writing, literature survey, and graphics of the manuscript. TN and DV contributed to technical intellectual content regarding data analysis, simulation, and imaging methods, and contributed to literature review and graphics. C-FW contributed to technical intellectual content regarding imaging and tractography. MH and PZ contributed to revising the manuscript with focus on clinical issues and contributed to the literature review. All authors contributed by revising the manuscript critically for important intellectual content.

## Conflict of Interest

KW is a shareholder of the biomedical optic spin-off company FluoLink AB. The remaining authors declare that the research was conducted in the absence of any commercial or financial relationships that could be construed as a potential conflict of interest.

## Publisher’s Note

All claims expressed in this article are solely those of the authors and do not necessarily represent those of their affiliated organizations, or those of the publisher, the editors and the reviewers. Any product that may be evaluated in this article, or claim that may be made by its manufacturer, is not guaranteed or endorsed by the publisher.

## Footnotes

1
https://liu.se/en/article/ne-downloads

## Abbreviations

AC-PC: anterior and posterior commissure
AI: artificial intelligence
cZi: caudal zona incerta
CT: computer tomography
DBS: deep brain stimulation
DRS: diffuse reflectance spectroscopy
DRT: dentato-rubro-thalamic tract
DWI: diffusion weighted imaging
EF: electric field
ET: essential tremor
FEM: finite element method
GPi: globus pallidus internus
LDF: laser Doppler flowmetry
LFP: local field potential
LiU: Linköping University
MER: microelectrode recording
ML: machine learning
MRI: magnetic resonance imaging
PD: Parkinson’s disease
PES: peri electrode space
PSA: posterior subthalamic area
STN: subthalamic nucleus
ROI: region of interest
UPDRS: Unified Parkinson’s Disease Rating Scale
VIM: nucleus ventrointermedius
VNA: volume of neural activation
VTA: volume of tissue activated.
